# Changes in CREB activation in the prefrontal cortex and hippocampus blunt ethanol-induced behavioral sensitization in adolescent mice

**DOI:** 10.3389/fnint.2013.00094

**Published:** 2013-12-13

**Authors:** Sabrina L. Soares-Simi, Daniel M. Pastrello, Zulma S. Ferreira, Mauricio Yonamine, Tania Marcourakis, Cristoforo Scavone, Rosana Camarini

**Affiliations:** ^1^Department of Pharmacology, Instituto de Ciências Biomédicas, Universidade de São PauloSão Paulo, Brazil; ^2^Department of Physiology, Instituto de Biociências, Universidade de São PauloSão Paulo, Brazil; ^3^Department of Clinical and Toxicological Analysis, Faculdade de Ciências Farmacêuticas, Universidade de São PauloSão Paulo, Brazil

**Keywords:** adolescence, behavioral sensitization, CREB, EMSA, mice

## Abstract

Drug dependence is a major health problem in adults and has been recognized as a significant problem in adolescents. We previously demonstrated that repeated treatment with a behaviorally sensitizing dose of ethanol in adult mice induced tolerance or no sensitization in adolescents and that repeated ethanol-treated adolescents expressed lower Fos and Egr-1 expression than adult mice in the prefrontal cortex (PFC). In the present work, we investigated the effects of acute and repeated ethanol administration on cyclic adenosine monophosphate (cAMP) response element-binding protein (CREB) DNA-binding activity using the electrophoretic mobility shift assay (EMSA) and the phosphorylated CREB (pCREB)/CREB ratio using immunoblotting in both the PFC and hippocampus in adolescent and adult mice. Adult mice exhibited typical locomotor sensitization after 15 days of daily treatment with 2.0 g/kg ethanol, whereas adolescent mice did not exhibit sensitization. Overall, adolescent mice displayed lower CREB binding activity in the PFC compared with adult mice, whereas opposite effects were observed in the hippocampus. The present results indicate that ethanol exposure induces significant and differential neuroadaptive changes in CREB DNA-binding activity in the PFC and hippocampus in adolescent mice compared with adult mice. These differential molecular changes may contribute to the blunted ethanol-induced behavioral sensitization observed in adolescent mice.

## Introduction

Epidemiological studies on drug consumption have revealed that ethanol is extensively consumed by adolescents (Degenhardt et al., 2008). Adolescents, compared with older subjects, are more sensitive to ethanol's appetitive effects and less sensitive to ethanol's aversive consequences (Spear and Varlinskaya, 2010). The adolescent period is a phase of neurochemical and neuroanatomical maturation in several brain regions (Spear, 2000). Developmental changes are observed in the hippocampus and prefrontal cortex (PFC) in adolescents compared with adults (Giedd et al., 1996; De Bellis et al., 1999), such as a decrease in glutamatergic synapses and an increase in dopaminergic synapses in both regions. These brain regions are particularly susceptible to the effects of alcohol in both human and rodent adolescents (De Bellis et al., 2000, 2005; Faria et al., 2008).

Among the major structures involved in motivational brain circuitry, the PFC has gained ground in the literature due to its role in drug abuse disorders. Ongoing maturation of PFC in adolescents and PFC function impairments have been associated with a greater risk of developing substance use disorders (Chambers et al., 2003). For instance, abstinent cocaine abusers show less activation of the lateral PFC (Bolla et al., 2004). Rats self-administer cocaine directly into mPFC, and this effect is attenuated by the lesion of dopamine neurons by 6-hydroxydopamine (Goeders and Smith, 1986). Piazza et al. (1991) also demonstrated that rats predisposed to self-administer amphetamine show reduced PFC dopaminergic activity. Dopamine in the PFC seems to be involved in behavioral inhibition and plays a pivotal role in motivational responses and impulse control (see Chambers et al., 2003 for review). A recent study showed that alcohol abstinence dysregulated the mPFC and central amygdala and induced impaired executive control over motivated behaviors, leading to an increase in alcohol drinking during acute abstinence (George et al., 2012). Furthermore, the medial PFC (mPFC) has been implicated in the behavioral sensitization processes (Beyer and Steketee, 2002). As examples, lesions of the mPFC disrupted the induction of sensitization to cocaine and amphetamine (Wolf et al., 1995; Cador et al., 1999; Li et al., 1999). The PFC is one of the last brain regions to reach complete maturation. An immature PFC is thought to be responsible for some of the typical behavioral characteristics of adolescents, such as poor judgment, risk behavior, and impulsivity (see Spear, 2000 for review).

Additional brain regions may also contribute for higher vulnerability of drug experimentation. PFC can modulate the activity of the other limbic areas (e.g., hippocampus) through glutamatergic projections. There is a clear connection between the PFC and the hippocampus. A direct pathway of communication from field CA1 of Ammon's horns to the infralimbic area of the PFC (Leichnetz and Astruc, 1975; Swanson, 1981; Férino et al., 1987) may represent the anatomical substrate of the relationship between PFC and hippocampus in mechanisms of learning and memory (Leichnetz and Astruc, 1975; Fuster, 1991). The prelimbic cortex is the PFC region where most of the hippocampal terminal fields are localized (Jay and Witter, 1991). For instance, glutamate signaling in the hippocampus is implicated in context-dependent sensitization to morphine (Xia et al., 2011). The hippocampus is involved in initiating and maintaining the reinforcing effects of morphine as well (Corrigall and Linseman, 1988; Self and Stein, 1993). In alcohol use disorders, the volume of hippocampus and PFC is seriously affected in adolescents (De Bellis et al., 2000, 2005).

Among the several models of neuroadaptation to drugs of abuse, behavioral sensitization has been correlated with the “incentive salience” of drugs and drug-associated stimuli (Robinson and Berridge, 2001), and it has been widely used by several alcohol researchers (Phillips and Shen, 1996; Quadros et al., 2003; Faria et al., 2008). Although sensitization to ethanol's effects is well established in adult mice, it is debatable whether adolescent mice sensitize to the stimulant effects of ethanol (Faria et al., 2008; Carrara-Nascimento et al., 2011). Ethanol treatment may induce changes in multiple target molecules, including immediate early genes (IEG). We found that adolescents developed context-dependent locomotor tolerance after repeated ethanol treatment at a low ethanol dose, accompanied by a suppression of Fos expression in the PFC and nucleus accumbens (Faria et al., 2008).

The transcription factor cyclic adenosine monophosphate (cAMP) response element binding protein (CREB) is a target of distinct signaling pathways and plays an important role in the addiction process (Carlezon et al., 2005). Among other signals, CREB can be phosphorylated at Ser-133 through a protein kinase A (PKA)-dependent mechanism. Phosphorylated CREB (pCREB) regulates the expression of many genes, including the activation of genes involved in drug addiction (Sheng and Greenberg, 1990; Asyyed et al., 2006). Ethanol induces the translocation of the PKA catalytic subunit into the nucleus (Dohrman et al., 2002), which appears to have long-lasting consequences. In this context, the PKA/pCREB signaling has been implicated in the molecular changes that underlie alcohol drinking and alcoholism (Dohrman et al., 1996; Asher et al., 2002; Pandey et al., 2005). Studies have investigated the effects of ethanol on pCREB (Yang et al., 1998; Pandey et al., 2005), but no study of which we are aware has demonstrated the molecular mechanisms that underlie CREB regulation in adolescent mice repeatedly treated with ethanol.

The present study focused on DNA-CREB binding activity in mice receiving acute and repeated ethanol during adolescence and adulthood. We assessed ethanol-induced sensitization and CREB activity. The rationale for using the behavioral sensitization model is that repeated drug administration in this protocol yields consistent age-related differences (e.g., Faria et al., 2008) and is associated with enduring neural changes (Robinson and Berridge, 2001). Our hypothesis is that DNA-CREB binding activity in mice repeatedly treated with ethanol during adolescence differs from the activity of adult mice.

## Materials and methods

### Animals and drug administration

Male Swiss mice [postnatal day (PND) 27–28 for the adolescent group and PND 65–70 for the adult group at the start of the experiments] were obtained from the Animal Facility of the Department of Pharmacology (ICB/USP, São Paulo, SP, Brazil). The animals were housed in groups of five in standard Plexiglas cages in a colony room with a 12/12 h light/dark cycle (lights on 6:30 AM–6:30 PM) and controlled temperature (22 ± 2°C). Food and water were provided *ad libitum*. The experiments were conducted during the morning phase between 8:00 AM and 12:00 PM. All animals care and procedures were approved by the Ethical Committee for Animal Research (CEEA) of the Institute of Biomedical Sciences, University of São Paulo. Ethanol (Merck do Brasil, Rio de Janeiro, RJ, Brazil) was dissolved in 0.9% w/v sodium chloride to produce a 20% v/v solution that was administered intraperitoneally (i.p) at a dose of 2.0 g/kg. Control animals received equivalent volumes of saline i.p.

### Apparatus

During each locomotor activity assessment, the animals were placed in the center of a cylindrical open-field arena (40 cm diameter × 35 cm height). A video camera installed 230 cm above the apparatus was connected to a computer located outside of the experimenter room. Horizontal locomotor activity was quantified for 10 min using EthoVision software (Noldus, Wageningen, The Netherlands) 5 min after a saline or ethanol injection.

### Blood ethanol concentration

To address the possibility that age could influence blood ethanol concentrations (BECs) via altered ethanol metabolism, a set of ethanol-treated animals (*n* = 6 per group) underwent behavioral sensitization procedure and was euthanized by decapitation immediately after the last behavioral test (15 min after the injection on day 22). A 0.5 ml blood sample was collected from each animal, and BECs were determined following procedures by Yonamine et al. (2003).

### Preparation of nuclear extracts

Nuclear extracts were prepared as described previously (Rong and Baudry, 1996), with minor modifications. Briefly, the PFC and hippocampus were scraped in lysis buffer and incubated on ice for 10 min. After the addition of NP-40 to a final concentration of 0.5%, the samples were vigorously mixed and centrifuged at 4°C for 30 s at 15,000 × *g*. The pellets that contained the nuclear fraction of interest were resuspended in extraction buffer, kept on ice for 20 min, and centrifuged at 4°C for 20 min at 15,000 × *g*. The resulting supernatants that contained nuclear proteins were aliquoted and stored at −80°C. The protein concentration was determined using the Bradford method (Bio-Rad).

### Immunoblotting of nuclear pCREB/CREB ratio

Electrophoresis was performed using 10% polyacrylamide in a Bio-Rad mini Protean III apparatus. Briefly, nuclear extracts (10 μg) were boiled at 95°C for 5 min, size-separated in 10% sodium dodecyl sulfate-polyacrylamide gels (100 V), transferred to a nitrocellulose membrane (Bio-Rad), and incubated with the following specific antibodies: anti-CREB-1 (Santa Cruz Biotechnology, Santa Cruz, CA, USA) at a dilution of 1:1000 or anti-pCREB (Millipore do Brazil, Barueri, SP, Brazil) at a dilution of 1:750 overnight under constant agitation at 4°C. To ensure equal protein loading, we used the Ponceau method to stain the membranes before probing with the antibodies. Afterward, the membranes were incubated with the secondary goat anti-rabbit antibody (Jackson ImmunoResearch Laboratories, West Grove, PA, USA) at a dilution of 1:10,000 for 1 h at room temperature under constant agitation. The proteins recognized by the antibodies were revealed by ECL Plus reagent following the manufacturer's instructions (Thermo Fisher Scientific, Waltham, MA, USA). β -actin antibody (Santa Cruz Biotechnology, Santa Cruz, CA, USA) was used as an internal control.

### Electrophoretic mobility shift assay

The electrophoretic mobility shift assay (EMSA) was used to analyze CREB using the gel shift assay kit from Promega. A ^32^P-CREB double-stranded consensus oligonucleotide probe (5′-AGAGATTGCCTGACGTCAGAGAGCTAG-3′; 20,0000 cpm) was incubated with nuclear extracts (10 μ g) in a binding reaction mixture for 30 min at room temperature. DNA-protein complexes were separated by electrophoresis through a 6% non-denaturing acrylamide: bis-acrylamide (37.5:1) gel in 0.5 ×Tris-borate/EDTA (TBE) for 2 h at 150 V. The gels were vacuum-dried, and autoradiographs were quantified using the ImageJ detection system (National Institutes of Health, Bethesda, MD, USA). Competition experiments were designed to demonstrate the specificity of the DNA-CREB complexes. An unlabeled CREB double-stranded consensus oligonucleotide was included in 5-, 10-, 15-, or 20-fold molar excess over the amount of the ^32^P-CREB probe to detect specific DNA-protein interactions. The unlabeled nuclear factor-κB (NF-κB; 5′-AGTTGAGGGGACTTTCCCAGGC-3′) double-stranded consensus oligonucleotide was included in 20-fold molar excess over the amount of the ^32^P-CREB probe to detect non-specific DNA-protein interactions. Supershift assays were designed to evaluate the components of the DNA-protein complexes using antibodies against CREB in its phosphorylated form (anti-pCREB; 1:10 and 1:20 dilution; Millipore) or against different CREB family components [CREB and cAMP response element modulator (CREM); 1:5, 1:10, and 1:20; Santa Cruz Biotechnology, Santa Cruz, CA, USA] according to each manufacturer's protocol. Data (arbitrary units) from the EMSA were expressed as an increased or decreased percentage of DNA-protein complexes compared with the control group (represented as 100%).

### Statistical analysis

The results are expressed as mean ± s.e.m. Behavioral data were evaluated through a Three-Way mixed analysis of variance (ANOVA; age × treatment × days), with days as the repeated measure. A Two-Way ANOVA (age × treatment) was performed to analyze test session data. Statistical comparisons for BEC, Western blot, and the EMSA were performed using ANOVAs, followed by the Newman-Keuls *post-hoc* test. All analyses were performed using Prism 4 software (GraphPad Software, San Diego, CA, USA). Alpha level was *p* < 0.05.

### Experimental procedures

#### Study of DNA-CREB complexes after acute ethanol treatment in adolescent and adult mice

This first part of the experiment sought to delineate the time-course of alterations in CREB activity after an acute locomotor-stimulating dose of ethanol in adult mice (2.0 g/kg) in nuclear proteins of the PFC and hippocampus. Briefly, the mice were allocated to five groups: Saline, Ethanol 1 h, Ethanol 3 h, Ethanol 6 h, Ethanol 24 h. On the first 2 days, the mice received i.p. saline injections and 5 min later underwent 10 min of exposure to the open field for the quantification of basal locomotor activity. On the third day, the mice were treated acutely with i.p. injections of saline or ethanol, and locomotor activity was quantified. Ethanol-treated animals were euthanized by cervical dislocation 1, 3, 6, and 24 h after the ethanol injection, and the PFC and hippocampus were dissected and stored at −80°C for the detection of DNA-CREB binding activity. Data from our laboratory revealed no difference in activation of CREB between 1 and 24 h in the control animals. For this reason, the saline group euthanized 1 h after the saline injection was used as a control. This data was processed to provide the ideal time to measure CREB activity in subsequent experiments with repeated ethanol treatment in adolescent and adult mice.

Two major DNA-CREB complexes were detected in the nuclear extract in the PFC and hippocampus. Another experiment was subsequently performed to identify putatives DNA-CREB complex composition in adolescent and adult mice, after they received saline or acute ethanol. The specificity of CREB binding was also evaluated. The mice were acutely treated with saline or ethanol according to the protocol described above and euthanized 3 h after the injection. The time of euthanasia (i.e., 3 h) was that in which we observed a decrease and increase in DNA-CREB binding activity after 2.0 g/kg ethanol in the PFC and hippocampus, respectively.

#### Study of the effects of acute and repeated ethanol injections on locomotor activity and DNA-CREB binding activity in adolescent and adult mice

Adolescent and adult mice were allocated to three groups: Saline-Saline (S-S; control group), Saline-Ethanol (S-Et; acute ethanol), and Ethanol-Ethanol (Et-Et; repeated ethanol). On the first 2 days (further referred as H1 and H2), all mice received a saline injection, and locomotor activity was assessed. The next day, they received either saline (S-S and S-Et groups) or ethanol (Et-Et group) during 15 consecutive days. Locomotor activity was measured on treatment days 1, 6, 10, and 15. One week after the last saline/ethanol injection (on day 22 of treatment), the control group received saline (S-S), whereas the acute ethanol and repeated-ethanol groups received ethanol (S-Et and Et-Et, respectively). Locomotor activity was evaluated 5 min after the saline or ethanol injection for 10 min. Three hours after the behavioral evaluation, the mice were euthanized by cervical dislocation, and the PFC and hippocampus were dissected and stored at −80°C for the EMSA assay, Western blot to determine CREB levels. The sensitization expression and the biochemical analysis were evaluated when mice previously treated with ethanol/saline during adolescence were PND = 51–52. The aim was to assess the behavioral and biochemical effects of ethanol in young adult mice pre-exposed to ethanol during adolescence. Thus, for these results, the adolescent mice will be referred as “adolescent group” (with this caveat in mind) or “mice pretreated with ethanol during adolescence.”

## Results

### Study of DNA-CREB complexes after acute ethanol treatment in adolescent and adult mice

**Figure 1:**
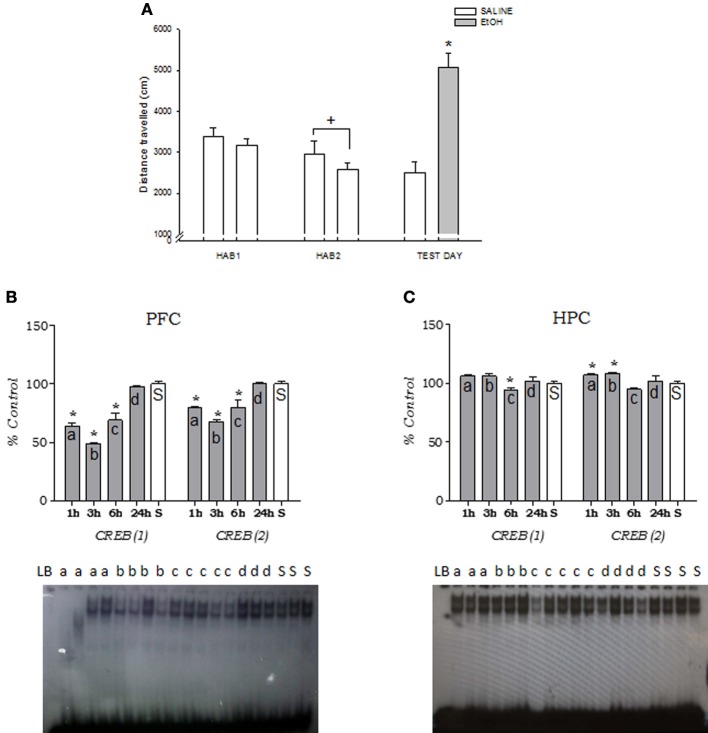
**(A)** Stimulant effects of an acute ethanol injection (2.0 g/kg) on locomotor activity in adult mice. Habituation H1 and H2 were analyzed using an ANOVA, followed by Newman-Keuls test; ^+^*p* < 0.05, H2 < H1. The data for the Test Day were analyzed using a *t*-test for independent samples; ^*^p < 0.05. Values are expressed as the distance traveled (cm) ± s.e.m. (SALINE *n* = 8; EtOH *n* = 20). **(B)** and **(C)** DNA-CREB binding activity at 1, 3, 6, and 24 h after an acute 2.0 g/kg ethanol injection in the **(B)** prefrontal cortex and **(C)** hippocampus. The data (expressed as a % from the control group) are expressed as mean ± s.e.m. (*n* = 5/group). A representative autoradiograph is shown. ^*^p < 0.05, different from the saline control group (S). LB = load buffer; S = saline.

Ethanol-treated adult mice displayed an increase in locomotor activity after 2.0 g/kg ethanol (*t* = 6.63, *p* < 0.05), confirming the stimulant effect of this low dose of ethanol (Figure 1A). Two days before the ethanol injection, the mice received a daily saline injection and were exposed to the open-field test (HAB1 and HAB2). The ANOVA (age × day) revealed a significant effect of day [*F*_(1, 26)_ = 14.9; *p* < 0.01], with a decrease in total locomotor activity at HAB2 compared with HAB1 (habituation effect).

The data of the temporal curve of DNA-CREB binding activity revealed two major bands that corresponded to CREB [labeled as CREB (1) and CREB (2)] that were quantified in all of the experiments (Figure 1). Ethanol significantly reduced DNA-CREB binding activity in the PFC at 1, 3, and 6 h, returning to similar levels as controls 24 h after the injection [One-Way ANOVA, CREB (1): *F*_(4, 20)_ = 76.09, *p* < 0.01;CREB (2): *F*_(4, 20)_ = 34.38, *p* < 0.01; Figure 1B]. In the hippocampus, we observed a decrease at 6 h for CREB (1) [*F*_(4, 20)_ = 14.82, *p* < 0.01] and an increase at 1 h and at 3 h for CREB (2) [*F*_(4, 20)_ = 13.36, *p* < 0.05; Figure 1C].

**Figure 2:**
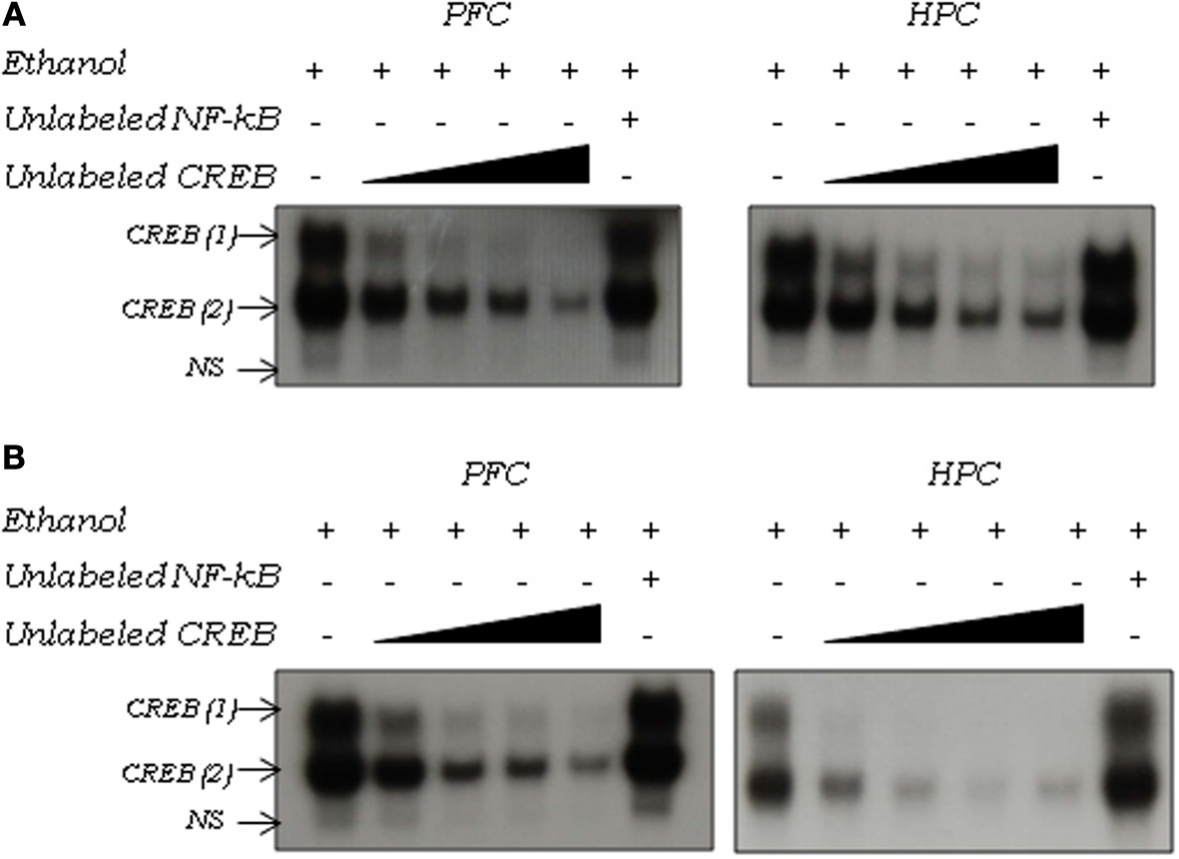
**Competition assay of CREB activation induced by ethanol in the prefrontal cortex (PFC) and hippocampus (HPC) in (A) adult and (B) adolescent mice using the EMSA**. Nuclear extracts (10 μg) were obtained 3 h after an acute 2.0 g/kg ethanol injection. Extracts were incubated in the absence (−) or presence (+) of 5- to 20-fold molar excess unlabeled specific (CREB consensus sequence) or 20-fold molar excess unlabeled non-specific (NF-κB consensus sequence) oligonucleotide. The positions of the DNA-CREB complexes [CREB (1) and CREB (2)] are indicated by arrows.

Figure 2 shows the two DNA-CREB complexes detected in the nuclear extract of the PFC and hippocampus in response to ethanol. In adult (Figure 2A) and adolescent (Figure 2B) mice, CREB (1) and CREB (2) was attenuated from 5-fold molar excess of unlabeled CREB oligonucleotide in both brain regions. In both ages and structures analyzed, a 20-fold molar excess of unlabeled NF-κB oligonucleotide did not inhibit DNA-CREB complexes, confirming the specificity of the DNA-CREB consensus sequence interaction.

**Figure 3:**
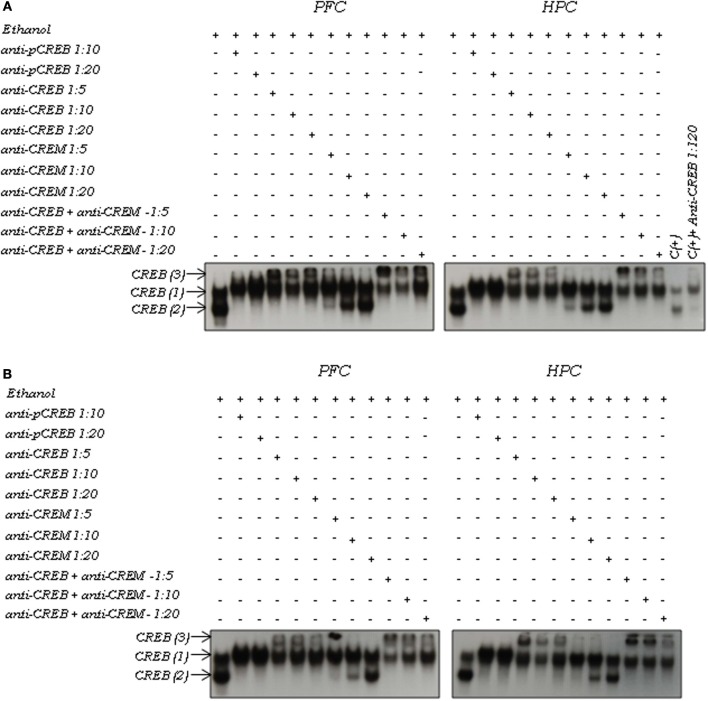
**Supershift assay of CREB activation induced by ethanol in the prefrontal cortex (PFC) and hippocampus (HPC) in (A) adult and (B) adolescent mice using the EMSA**. Nuclear extracts (10 μg) were obtained 3 h after an acute 2.0 g/kg ethanol injection. Extracts were incubated in the absence (−) or presence (+) of antibodies against pCREB (1:10 and 1:20), CREB (1:5; 1:10, and 1:20), and CREM (1:5; 1:10, and 1:20). The positions of the DNA-CREB complexes [CREB (1), CREB (2), and CREB (3)] and free probe are indicated by arrows. A nuclear extract was used as a positive control and also incubated with anti-CREB antibody (1:20).

Acute ethanol (2.0 g/kg) modulation of inducible DNA-protein complexes was analyzed in the nuclear extracts from the PFC and hippocampus in adult and adolescent mice using antibodies specific to members of the CREB family (CREB, pCREB, and CREM; Figure 3). No age or region differences were found in the composition of the DNA-protein complexes (described below).

Antibodies to the phosphorylated form of CREB caused a retardation of the mobility (supershift) of CREB (2) and possibly CREB (1). All DNA-CREB binding activity [CREB (1) and CREB (2)] was shifted using the anti-CREB antibody. The appearance of a third complex, named CREB (3), suggested a supershift of the CREB (1) complex. Complete inhibition of CREB (2) was observed in the presence of the higher concentration of anti-CREM antibody (1:5). However, the antibody to CREM did not influence the mobility of CREB (1). Antibodies to both CREB and CREM shifted CREB (1) and CREB (2). Again, the appearance of a third complex [CREB (3)] suggested a supershift of the CREB (1) complex. Therefore, CREB appeared to be the major component of the DNA-protein complexes, suggesting that CREB (1) is possibly a CREB homodimer and CREB (2) is a CREB-CREM heterodimer.

### Study of the effects of acute and repeated ethanol injections on locomotor activity and DNA-CREB binding activity in adolescent and adult mice

**Figure 4:**
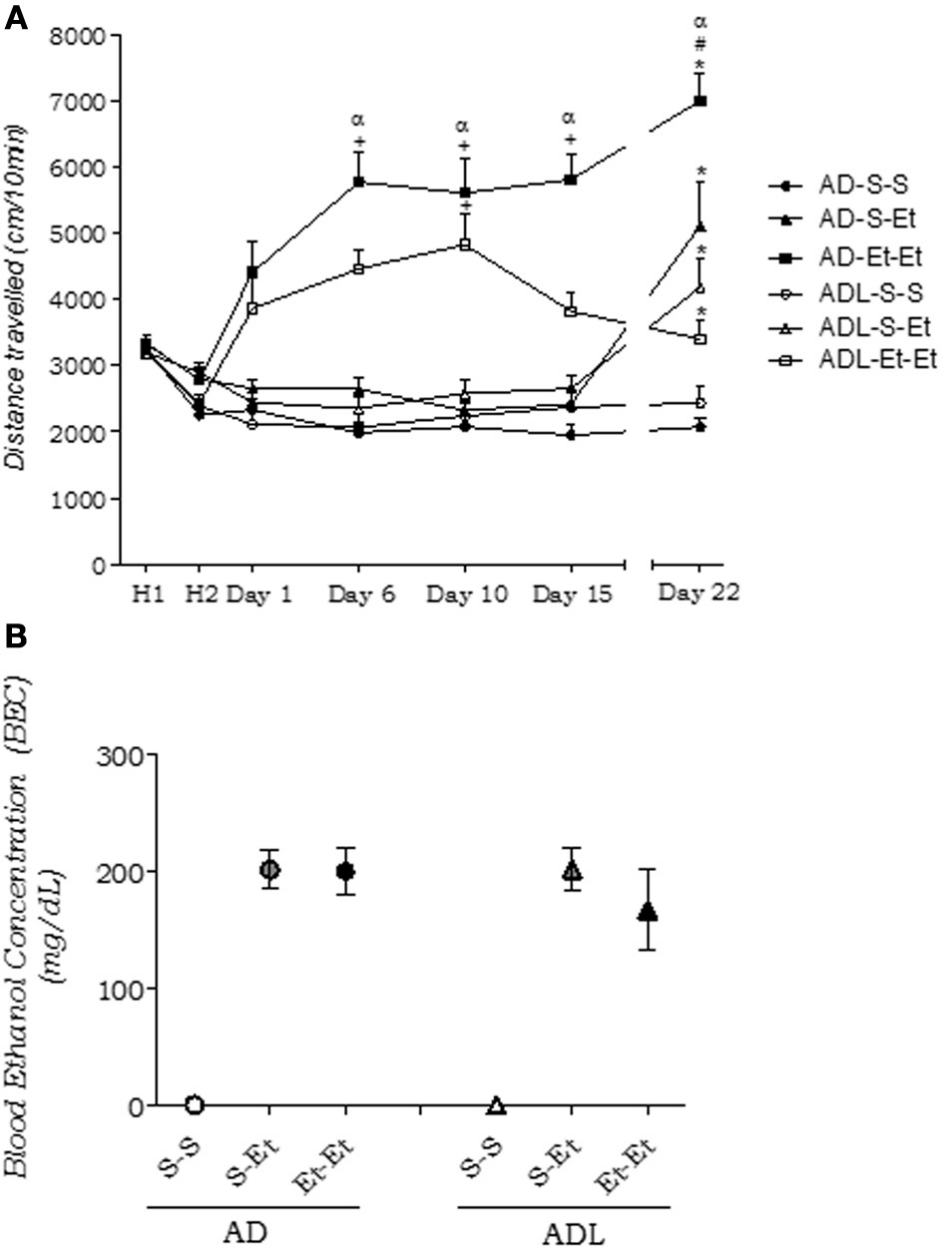
**(A)** Locomotor activity in adult (AD) and adolescent (ADL) mice treated repeatedly with 2.0 g/kg ethanol (Et-) or saline (S-) for 15 consecutive days (day 1–15). The graph also shows the expression of behavioral sensitization (day 22) following a saline (S-S) or ethanol challenge administration (S-Et and Et-Et). ^+^*p* < 0.01, different from the respective day 1 (Three-Way ANOVA for repeated measures followed by Newman-Keuls *post-hoc* test); ^α^*p* < 0.05, different from ADL-Et-Et group; ^*^p < 0.01, different from the respective control group (S-S; Two-Way ANOVA followed by Newman-Keuls *post-hoc* test); ^#^*p* < 0.05, different from the respective acute group. Values are expressed as the distance traveled (cm) ± s.e.m. (AD-S-S *n* = 17; AD-S-Et *n* = 17; AD-Et-Et *n* = 16; ADL-S-S *n* = 15; ADL-S-Et *n* = 14; ADL-Et-Et *n* = 17). ADL-S-S, adolescent control; ADL-S-Et, adolescent acute ethanol; ADL-Et-Et, adolescent repeated ethanol; AD-S-S, adult control; AD-S-Et, adult acute ethanol; AD-Et-Et, adult repeated ethanol. **(B)** Blood ethanol concentrations in adult (AD) and adolescent (ADL) mice on Day 22 after an ethanol challenge (*n* = 6/group). The data are expressed as mean ± s.e.m. S-S, control group; S-Et, acute ethanol group; Et-Et, repeated ethanol group.

The analysis of the results from day 1 to 15 using a Three-Way ANOVA with repeated measures (age × treatment × time) revealed an age × treatment × time interaction [*F*_(6, 270)_ = 3.32, *p* < 0.01; Figure 4A]. The *post-hoc* analysis showed that ethanol-treated adult mice developed locomotor activity sensitization in the second (day 6) exposure to the open field, unlike adolescent mice that did not sensitize. The analysis of the other factors confirmed a significant increase in locomotor activity in repeated ethanol-treated animals compared with the other groups [treatment factor: *F*_(2, 90)_ = 66.84, *p* < 0.01] and an age × treatment interaction [*F*_(2, 90)_ = 3.88, *p* < 0.05], demonstrating that adult mice showed greater locomotor activity compared with adolescent mice.

The Two-Way ANOVA performed on the data from day 22 of treatment (Figure 4A) revealed a significant age × treatment interaction [*F*_(2, 90)_ = 16.11, *p* < 0.01]. Repeated ethanol-treated adult mice showed the greatest locomotor activity compared with their respective controls and the acute ethanol groups, suggesting the expression of ethanol-induced locomotor sensitization. Adolescent mice repeatedly treated with ethanol displayed similar locomotor activity compared with their respective acute ethanol group, suggesting that they did not express sensitization. The locomotor activity values were 3401 ± 267 cm (Et-Et) *vs*. 4181 ± 421 cm (S-Et).

The BEC analysis showed no age-dependent differences [*F*_(1, 30)_ = 1.63; *p* > 0.05] (Figure 4B).

**Figure 5:**
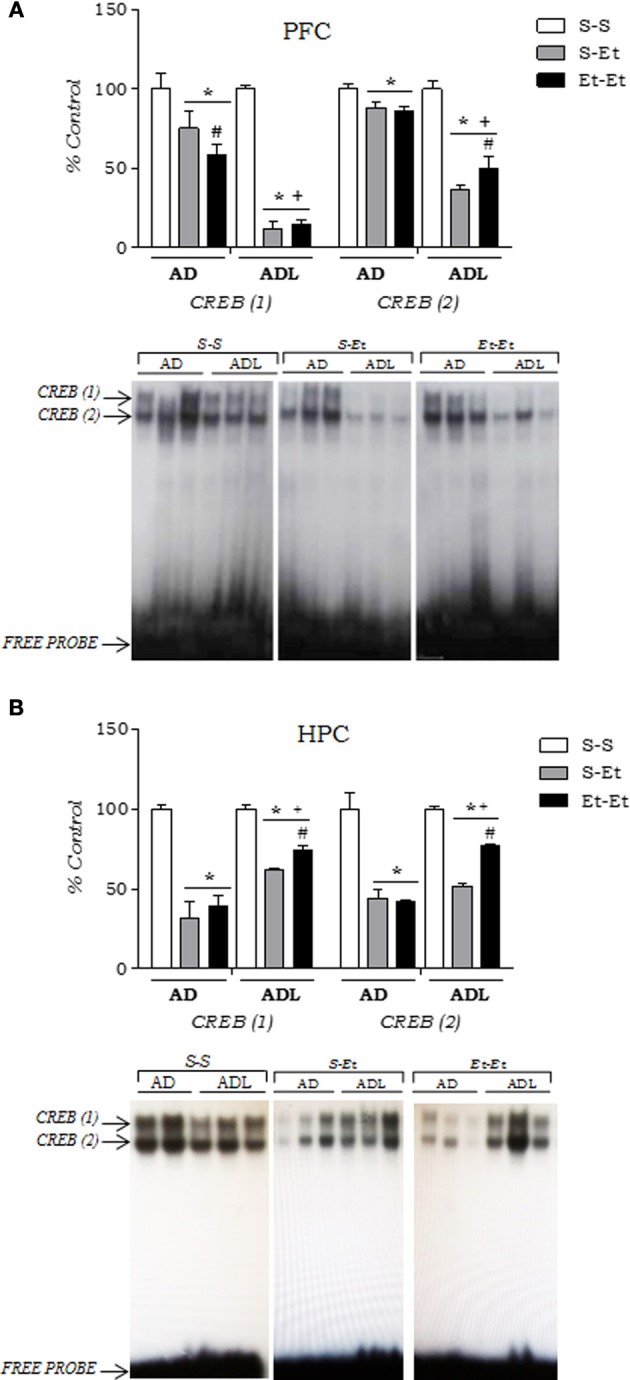
**CREB activation induced by acute or repeated ethanol treatment in the (A) prefrontal cortex (PFC) and **(B)** hippocampus**. An EMSA was performed with nuclear extracts (15 μg) from the PFC in adult (AD) and adolescent (ADL) mice assessed for behavioral sensitization to ethanol (2.0 g/kg), 3 h after the ethanol challenge (day 22); (*n* = 4/group). A representative autoradiograph is shown. Each bar represents the percentage (mean ± s.e.m.) of the results obtained in tissues from the respective control group (S-S). ^*^p < 0.01, different from the respective control group (S-S); ^+^*p* < 0.01, different from the respective treatment compared to the adult group; ^#^*p* < 0.01, different from the respective acute group (S-Et; Two-Way ANOVA followed by Newman-Keuls *post-hoc* test). S-S, control; S-Et, acute ethanol; Et-Et, repeated ethanol.

The effects of acute and repeated ethanol on DNA-CREB binding activity in the PFC and hippocampus are shown in Figure 5. A Two-Way ANOVA performed on the PFC data (Figure 5A) revealed an age × treatment interaction [CREB (1): *F*_(2, 30)_ = 12.1, *p* < 0.01; CREB (2): *F*_(2, 30)_ = 18.6, *p* < 0.01]. Although ethanol administration (2.0 g/kg, acute or repeated) reduced DNA-CREB binding [CREB (1) and CREB (2)] in the PFC [treatment factor for CREB (1): *F*_(1, 30)_ = 56.2, *p* < 0.01; treatment factor for CREB (2): *F*_(1, 30)_ = 42.4, *p* < 0.01] in both adolescent and adult mice, age-dependent differences were detected. Mice pretreated with ethanol during adolescence exhibited lower CREB-binding activity compared with adult mice [age factor for CREB (1): *F*_(1, 30)_ = 39.8, *p* < 0.01; age factor for CREB (2): *F*_(1, 30)_ = 64.9, *p* < 0.01]. Repeated ethanol induced lower CREB (1)-binding activity than acute ethanol in adult mice. The *post-hoc* analysis of the interaction also revealed an increase in CREB-binding activity [CREB (2)] after repeated ethanol treatment compared with the respective acute group in adolescent mice.

The analysis of the data from the hippocampus (Figure 5B) revealed an age × pretreatment interaction [CREB (1): *F*_(1, 12)_ = 6.01, *p* < 0.05; CREB (2): *F*_(1, 12)_ = 6.65, *p* < 0.05]. Ethanol administration (2.0 g/kg, acute or repeated) also decreased DNA-CREB binding [treatment factor for CREB (1): *F*_(1, 12)_ = 53.07, *p* < 0.01; treatment factor for CREB (2): *F*_(1, 12)_ = 59.34, *p* < 0.01] in both adolescent and adult mice, but unlike the results from the PFC, the reduction in DNA-CREB binding activity was greater in adult mice than in adolescent mice. The *post-hoc* analysis of the interaction also revealed an increase in CREB-binding activity [CREB (1) and CREB (2)] after repeated ethanol treatment compared with the respective acute group in adolescent mice.

**Figure 6:**
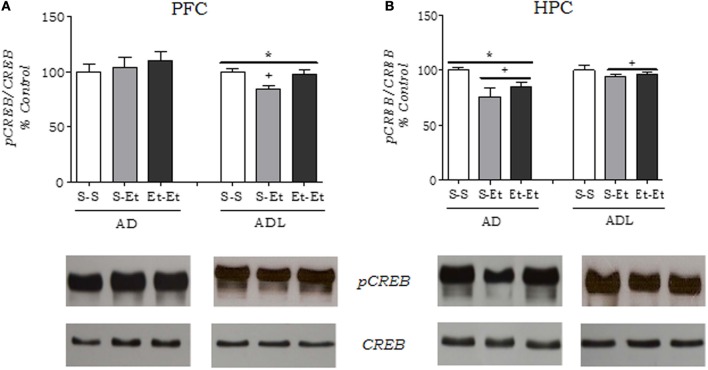
**Phosphorylated CREB levels assayed by Western blot in nuclear extracts of the (A) prefrontal cortex and **(B)** hippocampus in adult (AD) and adolescent (ADL) mice**. Membranes were stripped and reprobed for total CREB, and the final values are expressed as the pCREB/CREB ratio calculated for each sample. The values are expressed as mean ± s.e.m. (*n* = 6/group, randomly chosen from the groups used in the behavioral studies). The densitometric analyses of the results are represented below the graphs. Each bar represents the percentage of the results obtained in tissues in relation to the respective control group. ^*^p < 0.05, age-related differences; ^+^*p* < 0.05, different from the control group (S-S). (Two-Way ANOVA, followed by Newman-Keuls *post-hoc* test). S-S, control group; S-Et, acute ethanol group; Et-Et, repeated ethanol group.

Using a different set of mice, CREB and pCREB levels were also examined in the PFC and hippocampus by Western blotting (Figures 6A,B). A Two-Way ANOVA performed on the PFC data (Figure 6A) revealed an age effect [*F*_(1, 30)_ = 4.73, *p* < 0.05], with adolescent mice showing lower pCREB/CREB ratio than adult mice. In adolescent mice, the One-Way ANOVA revealed a decrease in the pCREB/CREB ratio in acute ethanol-treated mice compared with the respective control and repeated ethanol-treated mice [*F*_(2, 15)_ = 7.13, *p* < 0.01]. In adult mice, the One-Way ANOVA showed no differences among the groups [*F*_(2, 15)_ = 0.43, *p* > 0.05].

A Two-Way ANOVA performed on the hippocampus data (Figure 6B) revealed an age effect [*F*_(1, 30)_ = 8.023, *p* < 0.01], with adult mice showing lower pCREB/CREB ration than adolescent mice; and a treatment effect [*F*_(2, 30)_ = 6.4, *p* < 0.01], showing a reduction in pCREB/CREB ratio in acute and repeated ethanol groups compared to the saline control. In adolescent mice, a separate One-Way ANOVA showed no differences among the groups [*F*_(2, 15)_ = 0.81, *p* > 0.05]. In adult mice, the One-Way ANOVA revealed a decrease in the pCREB/CREB ratio in acute ethanol-treated mice compared with the respective control mice [*F*_(2, 15)_ = 5.33, *p* < 0.051].

## Discussion

The present study showed age-related responses to ethanol in CREB binding activity and also confirmed previous results from our laboratory, in which adolescent Swiss mice seem to be less sensitive to ethanol-induced behavioral sensitization than their adult counterparts (Faria et al., 2008; Camarini et al., 2010; Carrara-Nascimento et al., 2011, 2012). This appear to be related to changes in behavioral sensitivity rather than ethanol pharmacokinetics, since BECs in adolescent and adult mice were similar after acute and repeated ethanol treatment.

A significant reduction in the PFC, and an increase in the hippocampus, was observed in at least one CREB band between 1 and 3 h after ethanol administration. Ethanol stimulates specific isoforms of adenylyl cyclase (Yoshimura and Tabakoff, 1995), increasing the generation of cAMP and activation of PKA. Downstream responses of PKA activation include the phosphorylation of CREB and consequent induction of CREB-regulated genes, such as c-fos, c-jun (Sheng and Greenberg, 1990; Asyyed et al., 2006). pCREB binds to distinct consensus sequences, termed CRE (cAMP response element), in the promoter regions of genes regulated by the cAMP signaling pathway. We previously showed that Fos protein is rapidly expressed in the PFC and hippocampus after an acute ethanol injection in adolescent and adult mice (Faria et al., 2008). One could hypothesize that the expression of Fos after an acute ethanol injection may have occurred through the activation of PKA and subsequent CREB phosphorylation because Fos contains a CRE and is regulated by PKA (Sheng and Greenberg, 1990). In the present study, the alteration observed in CREB binding activity after acute ethanol was not accompanied by the same changes in Fos expression in the PFC. Indeed, the c-*fos* gene is not only modulated by PKA and CREB (Herdegen and Leah, 1998), and the number of Fos-containing neurons is not necessarily correlated with an increase in CREB phosphorylation (Turgeon et al., 1997). It is possible that CREB activation by acute ethanol might be correlated with Fos protein expression in the hippocampus but not in the PFC. Notably, acute ethanol increased CREB activity in the hippocampus in the first experiment (when mice received an acute ethanol injection after 2 saline injections), but decreased it in the second experiment (when mice received 17 saline injections before ethanol). This differential response seems to reflect the “stress” of the injections, and hippocampus is a brain region highly sensitive to stress (Kim and Diamond, 2002).

The competition assay validated the specificity of CREB to the consensus sequence, and the supershift assays suggested that CREB is the major component of DNA-protein complexes. CREB can form homodimers and heterodimers with CREM. It has been demonstrated that heterodimerization of CREB with other members of the Activating Transcription Factors (ATF) family decreases its stability and CRE binding affinity (Johannessen et al., 2004). The analysis of the CREB-specific band-shift complexes allowed us to suggest that one of the bands [CREB (1)] is possibly a CREB homodimer, whereas the other band [CREB (2)] is likely a CREB-CREM heterodimer. The composition of the DNA-protein complexes was similar between adolescent and adult mice and also between the PFC and hippocampus.

The main finding of this study was that acute and repeated ethanol decreased CREB binding activity in the PFC and hippocampus in both adolescent and adult mice but to different degrees. The suppression of CREB activity was more pronounced in the PFC of mice pretreated with ethanol during adolescence. The most marked reduction in the hippocampus was observed in adult mice. This result is consistent with our previous study (Faria et al., 2008), in which repeated ethanol administration desensitized the IEG regulated by CREB, Fos, in adolescent but not in adult mice in the PFC.

Adolescence is a period of remodeling and maturation in several brain structures. Specifically, the PFC is not completely mature during adolescence (Huttenlocher and Dabholkar, 1997; see Spear, 2000 for review). Dysfunctions in the PFC function are consistent with impairments in learning, organizational function, decision-making, risk-taking, and other behaviors, including a greater risk of developing addiction (Blumenfeld and Ranganath, 2007). Furthermore, addiction to substances of abuse is associated with impairment in decision-making (Bechara et al., 2001). Growing evidence indicates that a greater motivational drive for new experiences, associated with the immaturity of brain regions responsible for behavioral control, accounts for adolescents' higher vulnerability to drug experimentation (Casey et al., 2008). Altogether, the ongoing maturation of the PFC in adolescents is suggested to promote a state of impulsive attitudes and risky behaviors, including the use of addictive drugs. The PFC plays an important role in conflict situations and in tasks that require high-level cognition (Frith and Dolan, 1996), besides its role in the circuitry of addiction (Spear, 2000; Blumenfeld and Ranganath, 2007; Casey et al., 2008). Alterations in CREB function within the PFC are involved in cognitive function (Carlezon et al., 2005). Ethanol diminishes PFC firing *in vivo* (Tu et al., 2007) and affects several behaviors and responses that require neurons in the PFC, such as learning, information processing, working memory, and error detection, in humans and rodents (Melchior et al., 1993; Moselhy, 2001). Disruption of CREB function in the medial PFC impaired performance in the 5-choice serial reaction time task in rats (Paine et al., 2009), suggesting that decreased CREB function weakens attention in rats. Although we used a low dose of ethanol, one can predict that blunted CREB function in the PFC would induce changes in cognitive function. Thus, the possibility exists that a greater decrease in CREB binding activity in the PFC in ethanol-treated adolescent mice may reflect, at least partially, a failure in the cognitive control of appetitive cues. Our hypothesis is that the blunted ethanol behavioral sensitization seen in adolescent mice may be, at least partially, linked to an attenuation of ethanol-induced CREB activity in the PFC. The exact relationship between CREB activity in the PFC and sensitization remains to be determined. In agreement with the present results, chronic ethanol exposure was previously shown to decrease CREB-DNA binding activity and CREB phosphorylation in the rat striatum (Yang et al., 1998). Additionally, chronic ethanol treatment decreased the activity of adenylyl cyclase and PKA, reduced Gs protein expression and function, and increased Gi protein expression and function in the rodent brain (Valverius et al., 1989). Altered pCREB levels have been correlated with changes in the pattern of alcohol self-administration. Pandey et al. (2004) demonstrated that CREB-haplodeficient (±) mice have higher preference for ethanol than their wild type (+/+) littermates. This raises the hypothesis that decreased pCREB may causally be responsible for higher alcohol intake.

In contrast to the PFC, the reduction in DNA-CREB binding activity in the hippocampus was greater in adult mice than in adolescent mice. This result is consistent with findings of our laboratory that revealed desensitization of Fos expression in the hippocampus in adult mice but not in adolescent mice (Faria et al., 2008). These results suggest a reciprocal interaction between hippocampus and PFC, involving mechanisms for the counterbalanced regulation of memory-related transcription factors. In fact, an increase in levels of pCREB in the PFC during acquisition of conditioned place preference induced by intra-lateral hypothalamus stimulation was counteracted by a decrease in its levels in the hippocampus (Haghparast et al., 2011). The existence of a direct pathway from the CA1 region of the hippocampus to the infralimbic area of the PFC represents a link between those two brain regions in mechanisms of learning and memory (Leichnetz and Astruc, 1975; Swanson, 1981; Férino et al., 1987; Fuster, 1991).

Another age-related difference was the partial recovery to control levels of CREB (2) in the PFC and CREB (1) and CREB (2) in the hippocampus after repeated ethanol in adolescents, but not in adults, suggesting a neuroadaptive process. Consistent with these results, adolescents exhibited a total recovery of the pCREB/CREB ratio in the PFC. The pCREB/CREB ratio is not necessarily a mirror image of the levels of DNA-CREB binding activity, but it demonstrates the fraction of phosphorylated protein in relation to the total CREB signal. The phosphorylation of CREB at Ser-133 is affected by numerous kinases. Additionally, the binding of CREB to its target sequence (CRE) is determined by the transcriptional coactivator CREB-binding protein (CBP) (Carlezon et al., 2005). Still, the ratio pCREB/CREB reflects the results found in EMSA in which the effects of ethanol in the PFC were more pronounced in adolescents than in adults. In the hippocampus, this relationship was reversed.

Earlier exposure to ethanol decreases some of its aversive characteristics, such as bitter taste (Youngentob and Glendinning, 2009), capsaicin-like burning sensation (Glendinning et al., 2012), and increase subsequent ethanol intake (Pascual et al., 2009; Acevedo et al., 2010). Rodd-Henricks et al. (2002) also showed that alcohol-preferring P rats exposed to ethanol during adolescence displayed higher levels of responding for ethanol as compared to ethanol-naïve animals. Recently, we demonstrated a clear escalation in ethanol consumption in mice pretreated with ethanol during adolescence after a withdrawal phase but not in those pretreated with saline (Carrara-Nascimento et al., 2012). Altogether, these data demonstrate that exposure to ethanol during adolescence induces long-term behavioral effects.

In summary, the lower CREB function in the PFC of mice pretreated with ethanol during adolescence as compared to adults may have important implications for the higher vulnerability to ethanol addiction during adolescence or later in life.

## Author contributions

Rosana Camarini defined the research theme, designed the methods, interpreted the results and wrote the paper. Sabrina L. Soares-Simi carried out the laboratory experiments together with Daniel M. Pastrello and Zulma S. Ferreira, analyzed the data and helped to draft the paper. BECs were carried out by Mauricio Yonamine, Tania Marcourakis and Cristoforo Scavone analyzed the data and participated in drafting and revising the paper.

### Conflict of interest statement

The authors declare that the research was conducted in the absence of any commercial or financial relationships that could be construed as a potential conflict of interest.
